# Co-Operative Additive Effects between HLA Alleles in Control of HIV-1

**DOI:** 10.1371/journal.pone.0047799

**Published:** 2012-10-19

**Authors:** Philippa C. Matthews, Jennifer Listgarten, Jonathan M. Carlson, Rebecca Payne, Kuan-Hsiang Gary Huang, John Frater, Dominique Goedhals, Dewald Steyn, Cloete van Vuuren, Paolo Paioni, Pieter Jooste, Anthony Ogwu, Roger Shapiro, Zenele Mncube, Thumbi Ndung'u, Bruce D. Walker, David Heckerman, Philip J. R. Goulder

**Affiliations:** 1 Department of Paediatrics, University of Oxford, Oxford, United Kingdom; 2 eScience Group, Microsoft Research, Los Angeles, California, United States of America; 3 Nuffield Department of Medicine, University of Oxford, Oxford, United Kingdom; 4 Department of Medical Microbiology and Virology, University of the Free State/National Health Laboratory Services, Bloemfontein, South Africa; 5 Department of Internal Medicine, University of the Free State, Bloemfontein, South Africa; 6 Paediatric Department, University of Free State, Kimberley Hospital, Kimberley, Northern Cape, South Africa; 7 Botswana Harvard AIDS Institute Partnership, Gaborone, Botswana; 8 Department of Immunology and Infectious Diseases, Harvard School of Public Health, Boston, Massachusetts, United States of America; 9 HIV Pathogenesis Programme, The Doris Duke Medical Research Institute, University of KwaZulu- Natal, Durban, South Africa; 10 Ragon Institute of Massachusetts General Hospital, Massachusetts General Hospital, Boston, Massachusetts, United States of America; University of Alabama, United States of America

## Abstract

**Background:**

HLA class I genotype is a major determinant of the outcome of HIV infection, and the impact of certain alleles on HIV disease outcome is well studied. Recent studies have demonstrated that certain HLA class I alleles that are in linkage disequilibrium, such as HLA-A*74 and HLA-B*57, appear to function co-operatively to result in greater immune control of HIV than mediated by either single allele alone. We here investigate the extent to which HLA alleles - irrespective of linkage disequilibrium - function co-operatively.

**Methodology/Principal Findings:**

We here refined a computational approach to the analysis of >2000 subjects infected with C-clade HIV first to discern the individual effect of each allele on disease control, and second to identify pairs of alleles that mediate ‘co-operative additive’ effects, either to improve disease suppression or to contribute to immunological failure. We identified six pairs of HLA class I alleles that have a co-operative additive effect in mediating HIV disease control and four hazardous pairs of alleles that, occurring together, are predictive of worse disease outcomes (q<0.05 in each case). We developed a novel ‘sharing score’ to quantify the breadth of CD8+ T cell responses made by pairs of HLA alleles across the HIV proteome, and used this to demonstrate that successful viraemic suppression correlates with breadth of unique CD8+ T cell responses (p = 0.03).

**Conclusions/Significance:**

These results identify co-operative effects between HLA Class I alleles in the control of HIV-1 in an extended Southern African cohort, and underline complementarity and breadth of the CD8+ T cell targeting as one potential mechanism for this effect.

## Introduction

CD8+ T cells are a key component of the adaptive immune response to HIV-1, both in acute [1], [2] and chronic [3], [4] infection. This response is directed by the presentation of HIV-1 epitopes on the surface of infected cells by host HLA Class I molecules. The HLA-B locus is the strongest genetic determinant of disease outcome [5], [6], but beneficial effects of certain HLA-A [7], [8] and HLA-Cw [6], [9], [10] alleles have also been reported. Although a small number of disease-protective and disease-susceptible alleles have been well characterised, ascertaining the impact of many alleles can be difficult due to factors including low phenotypic frequency, linkage disequilibria between alleles, and small effects on disease outcome.

Based on these observations, and the known benefits of HLA Class I heterozygosity in mediating virologic control [11], we have recently investigated the potential for a co-operative additive effect between HLA alleles in suppressing viraemia, and demonstrated that certain combinations of alleles can work in tandem to mediate HIV-1 disease control [7], [12]. This effect is exemplified by HLA-A*74 and HLA-B*57 [7], alleles that occur in linkage disequilibrium in some Southern African populations, making the role of each individual allele on disease control potentially difficult to ascertain.

Larger cohorts allow for more refined analysis, enabling us to demonstrate that when each of two alleles independently exert a favourable impact, their co-occurrence may additionally have a combined effect. The test we have used here measures an effect where having two alleles working together additively has more impact on outcome (e.g. viral load or CD4+ T cell count) than having either one of them alone. This contrasts with a standard additive test which tests whether one allele has an additive effect above and beyond that of another. In the case where the first allele has little effect and the second allele a substantial effect, testing the two alleles against the first with a standard additive test would yield a positive result, whereas it would not with our test. We refer to the effect measured by our new test as a ‘co-operative additive effect’. The mechanism behind such effects is not clearly understood, but we have previously hypothesized that the reason for a combined benefit of HLA-A*74 and HLA-B*57 is – at least in part - the expanded repertoire of unique and complementary CD8+ T cell epitopes presented by the two alleles in combination [7].

We here built upon our previous methods [7] to further develop an extended systematic approach studying an enlarged Southern African cohort (Table 1). This aims to identify, first, the contribution of individual alleles to HIV-1 disease control, and second, any potential co-operative additive effects between pairs of HLA Class I alleles. We have generalized our previous method so as to allow identification of these effects irrespective of locus and linkage disequilibrium. We also sought to explore the hypothesis that these co-operative additive effects are accounted for by the enhanced breadth of CD8+ T cell epitopes presented by pairs of co-operative alleles, developing a new ‘sharing score’ to quantify breadth of unique CD8+ T cell responses, and demonstrating a correlation between breadth of responses and viraemic control.

**Table 1:** Characteristics of 2031 Southern African adult subjects with C-Clade HIV-1 infection enrolled in four cohorts.

Cohort Location	Number of Subjects	Median CD4 (IQR^a^), cells/mm^3^	Median VL (IQR^a^), copies/ml
Bloemfontein, South Africa	261	76 (32-548)	84000 (13000-250000)
Durban, South Africa	1225	376 (239-520)	38200 (7430-154500)
Kimberley, South Africa	31	326 (275-477)	47000 (8800-295000)
Gaborone, Botswana	514	342 (219-476)	19100 (3920-78200)
**Total**	**2031**	**352 (204-519)**	**32800 (6565-140454)**

a IQR = interquartile range.

## Results

### Univariate analysis confirms individual alleles that predict HIV-1 disease control or progression

We first sought to identify single HLA alleles that are predictive of better or worse disease outcome with respect to viral load and CD4+ T cell count in our cohort of 2031 Southern African adult subjects with C-clade HIV-1 infection. Using the more stringent cut-off of q<0.05 (FDR 5% [13]), we identified nine HLA alleles significantly associated with viraemic suppression (highlighted in Table 2, upper panel), and ten alleles associated with preservation of CD4+ T cell count (highlighted in Table 3, upper panel), representing a total of 14 different HLA class I alleles that are of benefit in disease control in this cohort (q<0.05). Five alleles, HLA-A*74, -B*57, -B*58:01, -B*81 and –Cw*18 were statistically associated with both lower viral load and higher CD4+ T cell count with q<0.05 (upper panels of Tables 2 and 3).

We also identified a total of nine different HLA alleles associated with a ‘hazardous’ (detrimental) outcome either with either respect to viraemic control (highlighted in Table 2, lower panel) and/or CD4+ T cell count (Table 3, lower panel) (q<0.05). Seven alleles were predictive of worse outcome with respect to both viral load and CD4+ T cell count: HLA-A*66, -B*08, -B*18, - B*45, -B*58:02, -Cw*06 and -Cw*16 (with q<0.05; lower panels of Tables 2 and 3).

**Table 2:** Univariate analysis of the impact of HLA Class I alleles on HIV-1 viral load in 2031 HIV-1 C-clade infected Southern African subjects (q<0.2).

HLA allele^a^	Phenotypic frequency (%)	Weight^b^	p-value	q-value
B*15:01	0.6	0.65	3.01E-02	0.11
B*57	6.2	0.63	8.42E-12	7.24E-10
Cw*12	3.2	0.45	4.48E-04	3.86E-03
A*74	10.2	0.34	1.03E-05	1.88E-04
B*13	2.8	0.32	1.96E-02	8.01E-02
B*39	3.6	0.32	7.44E-03	4.00E-02
Cw*18	11.6	0.29	2.49E-05	3.57E-04
B*81	10.0	0.28	1.60E-04	1.72E-03
B*58:01	10.6	0.26	3.81E-04	3.65E-03
A*33	4.8	0.23	3.12E-02	0.11
B*14	7.1	0.19	3.45E-02	0.12
B*42	22.8	0.18	1.12E-03	8.35E-03
A*01	7.2	0.17	5.23E-02	0.16
Cw*17	24.9	0.15	6.34E-03	3.63E-02
Cw*08	10.9	0.14	6.05E-02	0.16
Cw*04	23.6	0.14	1.33E-02	5.74E-02
B*44	16.6	0.13	3.13E-02	0.11
B*15:10	16.2	-0.11	7.00E-02	0.18
B*15:03	16.9	-0.11	6.01E-02	0.16
Cw*02	21.1	-0.12	3.68E-02	0.12
A*03	11.2	-0.13	6.00E-02	0.16
A*66	8.3	-0.21	9.31E-03	4.45E-02
Cw*16	11.6	-0.21	2.42E-03	1.60E-02
B*08	10.6	-0.23	1.54E-03	1.10E-02
A*68:01	6.4	-0.26	4.24E-03	2.60E-02
B*45	9.6	-0.30	1.42E-04	1.72E-03
Cw*06	29.4	-0.31	4.54E-10	1.30E-08
B*58:02	22.0	-0.35	9.71E-11	4.18E-09
A*80	1.6	-0.35	4.30E-02	0.14
B*18	6.1	-0.42	1.09E-05	1.88E-04

a Alleles in bold and underlined are those that remain significant with a more stringent FDR of q<0.05.

b Alleles with a positive weight (above double line) are associated with statistically significant disease control; alleles with a negative weight (below double line) are associated with hazardous (detrimental) outcome.

**Table 3:** Univariate analysis of the impact of HLA Class I alleles on CD4+ T cell count in 2031 HIV-1 C-clade infected Southern African subjects (q<0.2).

HLA allele^a^	Phenotypic frequency (%)	Weight^b^	p-value	q-value
B*40	0.5	0.28	3.55E-02	0.11
B*57	6.2	0.23	1.21E-08	7.96E-07
A*33	4.8	0.15	8.70E-04	6.38E-03
B*14	7.1	0.13	2.89E-04	3.28E-03
Cw*12	3.2	0.12	2.81E-02	9.28E-02
A*74	10.2	0.11	4.87E-04	4.60E-03
B*81	10.0	0.11	6.91E-04	5.70E-03
B*58:01	10.6	0.10	1.38E-03	7.60E-03
Cw*18	11.6	0.10	1.14E-03	7.49E-03
B*35	4.1	0.09	6.65E-02	0.19
Cw*04	23.6	0.09	6.04E-05	1.33E-03
Cw*08	10.9	0.08	1.04E-02	3.84E-02
B*44	16.6	0.07	4.93E-03	2.17E-02
A*30	36.3	-0.05	1.57E-02	5.44E-02
A*23	18.7	-0.05	4.44E-02	0.13
A*68:01	6.4	-0.07	7.04E-02	0.19
Cw*06	29.4	-0.08	1.70E-04	2.81E-03
A*66	8.3	-0.09	7.74E-03	3.01E-02
B*45	9.6	-0.10	4.00E-03	1.88E-02
B*08	10.6	-0.10	1.33E-03	7.59E-03
B*18	6.1	-0.11	5.59E-03	2.31E-02
Cw*16	11.7	-0.11	2.99E-04	3.28E-03
B*58:02	22.0	-0.12	9.20E-08	3.04E-06
B*41	1.9	-0.22	2.05E-03	1.04E-02

a Alleles in bold and underlined are those that remain significant with a more stringent FDR of q<0.05.

b Alleles with a positive weight (above double line) are associated with statistically significant disease control; alleles with a negative weight (below double line) are associated with hazardous (detrimental) outcome.

Using the less stringent criterion of q<0.2 (FDR 20%), we identified 17 alleles that are associated with favourable viraemic control and 13 alleles associated with poor viraemic control (Table 2). Likewise, for CD4+ T cell count with q<0.2, we identified 13 alleles associated with good outcome, and 11 alleles associated with lowered CD4+ T cell counts (Table 3). Many of these associations between HLA Class I alleles and HIV-1 disease outcome have previously been reported by ourselves and other groups studying C-clade infected cohorts [5], [7], [8], [14], [15], [16]. However, a previous univariate analysis in 1211 South African subjects demonstrated fewer alleles that are significantly associated with viral set-point (in this earlier work, only five such HLA associations remained significant after correction for multiple comparisons [12]). All five of these alleles previously reported to be significantly correlated with steady-state viral load feature again here in the extended list generated from analysis of an enlarged cohort (Table 2).

### Identification of pairs of HLA alleles that co-operate to influence HIV-1 disease outcomes

We identified six pairs of protective alleles that have a co-operative additive effect in mediating disease control (with q<0.05); these pairs are highlighted in Table 4. Three of these six HLA pairs that are associated with disease control are in significant linkage disequilibrium (that is, the two alleles in the pair are in linkage disequilibrium). Based on the computational method used, which accounts for linkage disequilibrium, linkage between alleles does not drive these results. That is, if two alleles are either in complete linkage, or are never observed together, then our test yields no statistical power to detect a co-operative additive effect, because the test needs enough examples of alleles to observe together, and apart, in order to assess the impact of having both as compared to just one. That two alleles arise together more frequently together than expected by chance cannot alone drive the test statistic. The enhanced size of this current cohort allowed us to identify many more associations than were previously described [12]; in fact, of all the pairs of alleles we here identified to have a beneficial effect on disease outcome, only the HLA-A*74/HLA-B*5703 combination was previously noted to impact favourably on viraemic control [12]. Another two allele pairs that were earlier reported to mediate a co-operative effect on disease outcome, HLA-B*81/HLA-Cw*04, and HLA-B*39/HLA-Cw*12 [12], did not reach statistical significance in this current analysis (i.e q>0.2 in each case).

**Table 4:** Multivariate analysis of pairs of HLA Class I alleles, showing pairs associated with favourable impact on HIV-1 disease control, with respect to maintenance of CD4+ T cell count and/or viral load suppression (q<0.2).

HLA-1^a^	HLA-2	Frequency of pair (%)	Linkage^b^(p-value)	Weight of pair	p-value for pair	q-value for pair
A*01	B*58:01	0.6	N/S	1.18	2.40E-03	1.91E-01
Cw*08	Cw*18	0.9	N/A	1.03	3.12E-03	1.98E-01
A*02	B*81	1.5	N/S	0.87	2.00E-05	2.97E-03
B*44	Cw*04	8.2	6.95E-33	0.84	2.00E-05	2.97E-03
A*74	B*57	1.5	1.75E-05	0.83	2.00E-05	3.71E-03
A*02	B*44	3.2	N/S	0.70	2.58E-03	1.91E-01
A*74	B*81	1.0	N/S	0.59	2.00E-05	3.71E-03
B*42	B*44	2.2	N/A	0.32	2.46E-03	1.76E-01
B*44	Cw*17	2.6	1.56E-05	0.28	2.74E-03	1.76E-01
B*14	B*81	0.6	N/A	0.25	8.20E-04	9.46E-02
B*14	Cw*18	0.4	N/S	0.24	4.20E-04	6.23E-02
B*58:01	B*81	0.8	N/A	0.21	2.00E-05	4.95E-03
B*58:01	Cw*04	1.2	9.33E-06	0.21	2.00E-05	4.95E-03
B*44	Cw*12	0.3	N/S	0.20	9.60E-04	9.46E-02
B*81	Cw*08	1.3	N/S	0.18	5.32E-03	2.00E-01
B*44	Cw*18	1.1	N/S	0.18	3.88E-03	2.00E-01
A*02	Cw*18	1.7	N/S	0.13	2.92E-03	1.97E-01
A*26	A*34	0.3	N/A	0.09	1.32E-03	1.09E-01

a Pairs in bold and underlined are those that remain significant with a more stringent q<0.05.

b Linkage is reported if relevant; otherwise designated as N/S = not significant (corrected for multiple comparisons; only values p<1.9E-05 are reported), or N/A = not applicable (two alleles at same locus).

Using the same approach, we also detected four ‘hazardous’ pairs of alleles with q<0.05 for which the expression of both alleles predicts worse disease outcome than expression of either one alone (these pairs are highlighted in Table 5). All pairs of alleles mediating a significant co-operative additive effect (with the less stringent FDR of q<0.2) are shown in Table 4 (beneficial pairs) and Table 5 (hazardous pairs).

**Table 5:** Multivariate analysis of pairs of HLA Class I alleles, showing pairs associated with hazardous (detrimental) impact on HIV-1 disease control with respect to decline in CD4+ T cell count and/or increased viral load (q<0.2).

HLA-1^a^	HLA-2	Frequency of pair (%)	Linkage^b^ (p-value)	Weight of pair	p-value for pair	q-value for pair
B*58:02	Cw*16	1.4	N/S	−1.17	2.00E-05	2.97E-03
B*45	B*58:02	1.3	N/A	−1.03	6.80E-04	7.21E-02
Cw*06	Cw*16	1.9	N/A	−0.93	2.00E-05	2.97E-03
Cw*02	Cw*06	3.7	N/A	−0.71	1.12E-03	1.04E-01
B*45	B*58:02	1.3	N/A	−0.68	2.60E-04	1.98E-01
B*18	Cw*16	0.5	N/S	−0.66	1.30E-03	1.38E-01
B*45	Cw*06	3.2	N/S	−0.57	2.84E-03	1.76E-01
A*68:01	Cw*02	1.3	N/S	−0.37	2.48E-03	1.76E-01
A*68:01	B*15:03	1.1	N/S	−0.37	2.58E-03	1.76E-01
A*23	B*41	0.6	N/S	−0.26	5.90E-03	2.00E-01
A*23	B*15:03	4.0	N/S	−0.23	4.00E-03	1.98E-01
B*08	B*18	0.4	N/A	−0.21	2.00E-05	4.95E-03
B*18	B*45	0.4	N/A	−0.21	1.24E-03	4.82E-02
B*15:10	B*58:02	2.4	N/A	−0.17	4.30E-03	2.00E-01
A*66	Cw*06	5.2	9.15E-22	−0.13	4.36E-03	2.00E-01
A*30	A*68:01	1.1	N/A	−0.13	5.12E-03	2.00E-01
B*15:10	Cw*06	3.0	4.02E-07	−0.13	3.82E-03	2.00E-01

a Pairs in bold and underlined are those that remain significant with a more stringent q<0.05.

b Linkage is reported if relevant; otherwise designated as N/S = not significant (corrected for multiple comparisons; only values p<1.9E-05 are reported), or N/A = not applicable (two alleles at same locus).

### Alleles that mediate a co-operative additive effect to control disease target a greater breadth of the HIV proteome

We hypothesized that co-operative additive effects in disease control might hinge on the presentation of combinations of alleles that present distinct epitopes from each other [9], [17], [18], as previously suggested for HLA-A*74 and HLA-B*57 [7]. Using the approach of calculating a ‘sharing score’ to quantify breadth of epitope coverage, as described in Materials and Methods, we demonstrated a significant correlation between the sharing score and the p-value of an additive effect for VL (R = −0.08, p = 0.03; data not shown). The direction of this correlation is in the expected direction, i.e. the negative R-value demonstrates that a larger sharing score (reflecting a greater breadth of epitope coverage) correlates with a smaller p-value (indicative of a stronger co-operative additive effect between alleles); thus, the greater the epitope coverage, the more the co-operative effect.

This suggests that some of the co-operative additive effect mediated by a pair of alleles can be accounted for by an increased breadth of CD8+ T cell targeting as compared to either allele alone. The effect was in the same direction (that is, a direction in which less sharing is correlated with being more co-operative, as expected), but not statistically significant, for CD4+ T cell count (R = −0.05, NS; data not shown).

## Discussion

These studies provide a useful resource in identifying HLA Class I alleles that mediate a co-operative additive effect in control of HIV-1 in C-clade infected African cohorts. The extended size of this cohort (>2000 individuals) and adaptation of methodology to identify co-operative additive effects has allowed us to build on previous analyses [7], [12] and to identify the impact of individual or paired HLA alleles with greater sensitivity. Importantly, however, in spite of this large cohort size, the analysis remains underpowered given the large number of HLA-pairs and the necessity of a multiple testing correction. These results are therefore likely an underestimate of the true extent of HLA co-operativity, and future studies employing more individuals, or a more restricted set of tests, are likely to reveal further instances of HLA co-operativity. Furthermore, our approach of using most HLA data at two-digit resolution was aimed to maximize statistical power to detect Class I influences on disease control. However, a caveat of this approach is that it limits the detection of possible differences occurring at high-resolution (often a micropolymorphism) level [19]; this could be addressed in future by use of larger cohorts.

Effects on disease control were not always seen for both CD4 count and VL. Reasons for this likely include imperfect correlation between CD4 count and VL (r^2^ = 0.22, p<0.0001 by linear regression; data not shown), and that the linear models are only idealizations.

Our analysis supports previous evidence that even highly beneficial responses, such as that restricted by HLA-B*57, can be improved upon by addition of other T cell responses [7], [12]. The mechanism of this phenomenon has not previously been clearly characterised, but we have here demonstrated that – at least in part - the effect may be explained by the targeting of non-overlapping CD8+ T cell epitopes across the HIV proteome.

The correlation between our ‘sharing score’ (reflecting breadth of epitopes targeted by a pair of alleles) and the probability of a co-operative additive effect mediated by these alleles was only weak (R = −0.08). Any computational method to assess breadth of epitope targeting is a challenge, especially given the density of overlapping CD8+ T cell epitopes in certain regions of the HIV proteome, the bias towards restricting highly targeted epitopes restricted by prevalent Class I alleles, and the complexity of immunodominance patterns. In addition, any single pair of alleles will also be impacted by the other four HLA Class I molecules expressed by a given individual, and the overall disease outcome will be influenced by many factors in addition to HLA genotype. Furthermore, there is no obvious effect size obtainable for the co-operative additive test, and even if there were it would be possible to have large effects for pairs which were not statistically significant. For these two reasons, we chose to measure correlation with the p-value from our test.

These difficulties notwithstanding, these data nevertheless do highlight that two alleles which present different epitopes can each confer a separate benefit (or hazard) to the individual; thus having both of them is better (or worse) than having just one of them and a co-operative additive effect is at play. However, if two alleles present many of the same epitopes (as exemplified by HLA-B*57 and -B*58:01, or HLA-B*42 and -B*81), they are less likely to act together co-operatively – having one of them may be little different from having both. This effect is also underscored by the phenomenon of heterozygote advantage [11], which may be mediated by increased breadth of epitopes presented by HLA class I heterozygotes compared to homozygotes.

As HLA-peptide complexes are ligands not only for T-cell receptors on CD8+ T cells, but also for KIR receptors on NK cells [20], [21], another potential reason for the favourable (or hazardous) interaction of some pairs of HLA alleles is the combined effect of a CD8+ T cell response and an NK-cell response. Homozygosity for KIR ligands may also explain poor disease outcomes in subjects with certain HLA Class I combinations, although many of our pairs involved at least one allele that is not a known KIR ligand.

Characterising interplay between HLA alleles is made difficult by the presence of linkage disequilibrium between alleles. However, our test statistic will not be significant for two alleles simply because they are in linkage disequilibrium, but rather the test can find two alleles to have a co-operative additive effect *despite* their being in (incomplete) linkage disequilibrium, albeit with reduced power owing to fewer observations of the alleles acting one without the other. That is, if one observes each allele only in the context of the other, or never together, it is impossible to determine whether nor not they have a co-operative additive effect (hence these pairs removed from analysis; see Methods section). However, because one needs to observe enough co-occurrences of the alleles, having alleles in incomplete linkage disequilibrium increases the power to detect co-operative additive effects.

In summary, these data highlight the potentially potent interactions between HLA class I alleles to mediate HIV-1 disease control. Even CD8+ T cell responses which are independently associated with strong viraemic suppression and sustained immunological control can be improved upon by the co-expression of certain other favourable HLA class I molecules. This finding underscores the potential benefit of harnessing co-operative effects of multiple CD8+ T cell responses in the development of CD8+ T cell vaccines.

## Materials and Methods

### Ethics statement

Ethics approval was given by University of KwaZulu-Natal Review Board and the Massachusetts General Hospital Review Board (Durban cohort), the Office of Human Research Administration, Harvard School of Public Health and the Health Research Development Committee, Botswana Ministry of Health (Gaborone cohort), and the University of the Free State Ethics Committee (Durban and Kimberley cohorts). All subjects provided written informed consent.

### Recruitment and characterization of patients

We recruited 2031 HAART-naïve, southern African adult subjects with chronic C-clade HIV-1 infection via four cohorts (Table 1): (i) Durban, South Africa [9], [12]; (ii) the Gaborone region, Botswana [22]; (iii) Bloemfontein, South Africa [23]; (iv) Kimberley, South Africa [7]. The exact timing of infection in each individual was not known, but all these subjects were either presenting with clinical features of HIV infection, or diagnosed by routine screening in pregnancy – in both cases, in keeping with chronic infection. Viral loads (VL) were obtained for 1873 subjects using the Roche amplicor 1.5 assay and CD4+ T cell counts were determined for 1871 subjects using flow cytometry. All subjects had either a viral load or a CD4+ count available for analysis; the majority (84%) had both. Although a single measurement of VL and CD4+ T cell count for each individual is a limited ‘snap-shot’ of disease, these parameters are known to correlate well with disease outcome/time to AIDS.

HLA typing was performed from genomic DNA by sequence-based typing. We collapsed all HLA data to two-digit HLA-types, with three exceptions in which the four-digit type is most likely to be critical to disease outcome: HLA-A*68:xx [12], [24], HLA-B*15:xx [12], [25], and HLA-B*58:xx [5], [12], [26]. An HLA imputation tool was used to infer those alleles not collapsed if they were only typed to two-digit level for any individual [27]. Data were removed for 75 subjects in whom the four-digit type for HLA-A*68:xx, HLA-B*15:xx or HLA-B*58:xx could not be determined.

Gag population sequences (p17+p24) were generated from genomic DNA for 1256 individuals, as previously described [9], [23].

### Univariate analysis of impact of HLA Class I alleles on HIV-1 disease control

We undertook a univariate analysis to assess the impact of individual HLA alleles on disease control. Such a scan has not always been applied in previous studies that have examined HLA associations with HIV viral setpoint or absolute CD4+ T cell count. As such, the contributions of HLA-A and HLA-Cw alleles that often have less impact than HLA-B have tended to be obscured [5], [7]. Disease control was defined as previously, using continuous-valued data (absolute CD4+ T cell count and absolute viral load) and discrete targets (‘controller’ defined as CD4+ T cell count >250 cells/mm^3^; viral load ≤2000 RNA copies/ml plasma) [7]. The univariate analysis was performed using an LRT test with linear or logistic regression for, respectively, continuous-valued and discrete targets (for example, CD4+ T cell count is real-valued, whereas ‘controller’ was the binary, thresholded version of CD4+ T cell count). We evaluated only HLA Class I alleles occurring at a phenotypic frequency of ≥0.5%, and used False Detection Rate (FDR) q<0.05 (5% false positive) or q<0.2 (20% false positive) [13].

### Multivariate analysis of impact of pairs of HLA Class I alleles on HIV-1 disease control

To identify any two HLA Class I alleles with co-operative additive effects on disease control, we used previously published methodology [7]. As described in the introduction, we used the term ‘co-operative additive’ to describe interplay between two alleles that is more than a simple additive effect. Briefly, each HLA combination was tested to see whether an additive model for two alleles together performed better in predicting disease outcome than a model that did not allow both alleles to interact. In contrast to previous analysis [7], we here generalized the test by removing the restriction that correlations need to be in the ‘direction of control’, allowing for detection of combinations of ‘beneficial’ and ‘hazardous’ alleles, or two ‘hazardous’ alleles. Note that P-values were computed for our test in a non-parametric way - using permutations. The test statistic for an HLA pair was the difference in log likelihood between these two models after fitting each by maximum likelihood. P-values were obtained by 50 K permutations of one HLA allele in the test [7].

As previously, correcting the analysis for cohort origin using cohort covariates was highly statistically significant [7], but richer lineage-correction using a linear mixed model (with a phylogenetic tree-based variance component using Gag sequences) provided no further benefit; therefore, cohort covariates alone were added to the analysis. In all paired analyses, we set two criteria for inclusion of a pair of alleles in the analysis (i) alleles must be expressed together in at least five subjects, and (ii) alleles must occur independently of one another in at least five subjects (thereby removing any pairs in near or complete linkage). The value five was chosen based on other similar work (e.g. Microsoft PhyloD, which routinely uses a ‘min count’ filter for the minimum number of times an HLA allele must appear [28], [29]). Because this filtering step does not consider VL or CD4+ T cell count values, it is a statistically valid approach, and is conservative in that it can only cause us to miss real associations, not to detect false associations spuriously (regardless of the actual filtering threshold used). Specifically, this filtering threshold was not manipulated in response to the data (we only ever used this one threshold). Such ‘min count’ thresholds are widely used in similar contexts – e.g. all genome-wide association studies where mean allele frequency and Hardy-Weinberg equilibrium thresholds are employed as a preprocessing step (for example, see [30]).

### Statistical tests

Linkage disequilibrium between HLA class I alleles was computed using Fisher's Exact Test using the on-line tool at the Los Alamos HLA molecular immunology database: http://www.hiv.lanl.gov/content/immunology/hla/hla_linkage.html. This method reports significant linkage following correction for the number of tests performed (in this case, threshold for significance is p<1.9E-05).

Statistical correction for multiple comparisons was performed using a False Discovery Rate (FDR) with thresholds of q<0.05 (5% FDR) or q<0.2 (20% FDR) [31].

### Methods to identify correlation between breadth of epitope targeting and disease control

In order to investigate any relationship between HIV-1 disease control (mediated by any pair of HLA alleles) and breadth of CD8+ T cell responses, we used IFN-g ELISpot data for 1010 South African subjects tested against a panel of 410 C-clade overlapping peptides (OLPs) spanning the entire HIV-1 proteome, as previously described [17], [32]. We first assigned likely HLA allele restriction(s) to each OLP using stepwise Fisher's Exact Test (FET) to control for linkage disequilibrium. In each iteration, the most significantly associated HLA allele was determined using FET, then all individuals who expressed that allele were removed and the next most significant allele (with corresponding p-value) was identified. All alleles associated with the OLP at q<0.2 [33] were considered restricting alleles. For each pair of alleles, we computed a ‘sharing score’ as a means of quantifying the breadth of unique epitopes targeted by any given HLA pair. This sharing score was calculated as the number of shared OLPs divided by the number of unique OLPs targeted by the pair. Thus a higher sharing score indicates less total breadth of epitope coverage across the proteome.

For each pair of alleles, this sharing score was correlated with the p-value from the additive pairs analysis using a Pearson correlation. We confirmed the analytically-computed P-values yielded by Pearson by using a permutation test with 1000 permutations, and the P-values from both approaches were in agreement.

Note that there is no obvious effect size obtainable for the co-operative additive test, and even if there were it would be possible to have large effects for pairs which were not statistically significant. For these two reasons, we chose to measure correlation with the p-value. Based on this approach, a larger sharing score (indicative of wider OLP targeting) correlating negatively with the p-value for an additive effect (where a smaller p value is indicative of a stronger co-operative additive effect between alleles) points to a relationship between breadth of coverage and two alleles acting co-operatively toward immune control.
